# Design and imaging performance of BPET–DBT – a dedicated scanner for breast imaging

**DOI:** 10.1002/mp.70651

**Published:** 2026-09-14

**Authors:** Srilalan Krishnamoorthy, Emmanuel Morales, William J. Ashmanskas, Matthew E. Werner, Samuel Matej, Juhi Raj, Trevor L. Vent, Chloe J. Choi, Andrew D. A. Maidment, David A. Mankoff, Christine E Edmonds, Joel S Karp, Suleman Surti

**Affiliations:** ^1^ Department of Radiology University of Pennsylvania Philadelphia Pennsylvania USA; ^2^ Department of Physics and Astronomy University of Pennsylvania Philadelphia Pennsylvania USA; ^3^ Department of Bioengineering University of Pennsylvania Philadelphia Pennsylvania USA; ^4^ Abramson Cancer Center University of Pennsylvania Philadelphia Pennsylvania USA

**Keywords:** BPET‐DBT, Dedicated breast PET, TOF‐PET

## Abstract

**Background:**

A dedicated breast PET (BPET) scanner integrated with digital breast tomosynthesis (DBT) enables three‐dimensional functional imaging with quantitative information co‐registered to high‐resolution anatomical images. With the availability of breast cancer specific PET radiotracers, such a scanner has the potential to improve treatment planning in breast cancer. We recently developed a BPET–DBT scanner utilizing an integrated gantry design to generate intrinsically co‐registered BPET–DBT images.

**Purpose:**

This paper presents the scanner design, characterizes its imaging performance, and reports on our initial human imaging experience to demonstrate scanner's clinical readiness.

**Methods:**

The PET component is comprised of two detector heads, providing a 20 × 10 × 10 cm^3^ scanner field‐of‐view. The detector provides time‐of‐flight (TOF) measurement and is comprised of 32×32 LYSO arrays of 1.5 × 1.5 × 15 mm^3^ crystals coupled to multi‐anode PMTs. Custom waveform‐sampling electronics ensures high spatial and timing resolution with minimal deadtime. The DBT component is a state‐of‐the‐art system which employs 2D x‐ray tube and detector motion for improved contrast and resolution. Phantoms appropriate for breast imaging were imaged to assess spatial resolution, image quantitation, count‐rate capability, and co‐registration accuracy. First human imaging was performed in volunteers with estrogen‐receptor‐positive (ER+) breast cancer. A five‐minute BPET–DBT scan using ^18^F‐fluoroestradiol was acquired and compared with a whole‐body PET scan.

**Results:**

System timing resolution of 425 ps and energy resolution of 16% were measured. Spatial resolution measurements showed an in‐plane (parallel to the detector) resolution of 2 mm and demonstrates the ability to resolve 1.6 mm rods in the micro‐deluxe hot rod phantom. TOF reconstruction combined with image‐based resolution modeling mitigates artifacts typical in scanners with incomplete angular coverage and enables good out‐of‐plane (orthogonal to detector) discrimination of the 2.4 mm rods. Count‐rate measurements confirm low scanner dead‐time, with sufficient count‐rate capability for clinical use.

**Conclusions:**

We successfully constructed and tested a BPET scanner and integrated it with DBT. BPET scanner performance was characterized, and imaging capabilities relevant for clinical imaging have been highlighted. Images from a patient with ER+ breast cancer show clear tumor delineation and visualization of heterogeneous uptake, as also seen with WB‐PET. This approach offers a promising strategy for improved treatment planning and outcomes in breast cancer care.

## 1 INTRODUCTION

Breast cancer is the most common malignancy among women, recently surpassing lung cancer.^1^ It is a heterogeneous disease with multiple molecular subtypes, each having varying behavior and prognosis. Currently treatment options are increasingly personalized and often use receptor status to guide treatment selection and assess treatment response. Therefore, diagnostic tools that can both detect cancer and provide biological insights could greatly enhance the effectiveness of prevailing treatment strategies.

PET imaging for breast cancer is currently most commonly performed using whole‐body PET (WB‐PET) scanners that have spatial resolution of 3–5 mm.^2^, ^3^, ^4^, ^5^ It therefore remains challenging for WB‐PET to accurately quantify lesion uptake in smaller lesions (<5 mm) that are prevalent in early‐stage breast cancer. Several dedicated breast PET scanners have been developed over the past three decades to improve upon the limitations of using WB‐PET in breast imaging. These efforts were primarily driven by academic and research institutions that developed these dedicated breast PET scanners, demonstrated their clinical benefits in detecting <1 cm lesions,^6^, ^7^, ^8^, ^9^, ^10^, ^11^, ^12^, ^13^, ^14^, ^15^, ^16^, ^17^ and eventually paved the way for the commercial development of dedicated breast PET scanners.^18^, ^19^, ^20^, ^21^, ^22^, ^23^ These scanners were primarily developed to improve tumor detection with higher spatial resolution. However, the recent development of novel radiotracers (e.g. ^18^F‐FES, ^18^F‐MISO, ^18^F‐FLT, ^68^Ga‐FAPI) capable of tumor characterization has the potential to expand the role of breast PET beyond cancer detection.

We have built a high spatial resolution, dual‐panel, time‐of‐flight breast PET scanner (BPET) that is combined with a digital breast tomosynthesis (DBT) scanner in an integrated gantry to provide intrinsically co‐registered BPET–DBT images. The primary rationale for employing a dual‐panel PET scanner design is its enhanced clinical utility through seamless integration into the existing clinical mammography and tomosynthesis workflow which often involves breast tissue biopsy. Besides providing anatomical information, the DBT image also facilitates accurate attenuation correction of the PET imaging and is necessary for accurate lesion quantification. The BPET–DBT scanner can thus provide quantitative 3D images of relevant tumor biomarkers co‐registered with a high‐resolution anatomical image. Beyond breast cancer detection, this device has the potential to contribute to several diagnostic and treatment planning applications within the context of personalized breast cancer management.^24^ These applications include assessing extent of local disease, performing in vivo tumor biology characterization to guide therapy selection and evaluate treatment response and delineate tumor boundaries. In comparison with conventional dual‐panel PET scanners that suffer from image artifacts and reduced quantitation accuracy due to limited angle effects, time‐of‐flight (TOF) information reduces these effects and improves image quality and consequently accuracy of lesion quantification.^25^, ^26^ This paper describes the design of the hybrid BPET‐DBT system, presents a detailed physical characterization and imaging performance of the PET component, and a first patient study performed with the hybrid device. In the absence of standardized evaluation protocols, the performance evaluation was based on standard NEMA NU2 and NU4 methods^27^, ^28^ for whole‐body and small animal scanners respectively, that were adapted for application to a dedicated breast PET scanner. These evaluations were also supplemented with customized tests designed to reflect the intended clinical use of the BPET scanner.

## 2 MATERIALS AND METHODS

### 2.1 BPET – scanner design, data acquisition and image reconstruction

The BPET scanner is comprised of two detector heads, each with a 4 × 2 array of PET detectors covering a field‐of‐view (FOV) of 20 cm × 10 cm. The PET detector employs a 32×32 array of 1.5 × 1.5 × 15 mm^3^ LYSO crystals read out by the Hamamatsu H8500 PMT.^29^ The detector head separation is variable but was fixed at 11 cm for this work. This provides a scanner FOV of 20 × 10 × 10 cm^3^ to accommodate the anatomy of large majority of the US adult female population. Scanner geometry simulations demonstrated the need for high TOF resolution and adequate geometrical coverage to mitigate the effects from limited‐angular coverage, to maintain good image quality and quantitative accuracy.^26^ Custom waveform‐sampling data acquisition (DAQ) electronics (ROCSTAR),^30^ based on the commercially available DRS4 chip,^31^ was developed specifically for this BPET scanner. ROCSTAR has a modular design, with a single board to readout a single detector, and samples waveforms at 3.5 Gsps to preserve the detector timing resolution at the system level. A separate custom board called Master Coincidence/Control Unit (MCU) was used to synchronize all ROCSTAR boards, identify coincidences in real‐time, and also read‐out coincidence events using a ±2.2 ns coincidence window. The prototype MCU used for this work supports communication with only 8 BPET detectors. Consequently, the BPET scanner used in this study had an electronically active FOV of 5 cm along the anterior‐posterior direction (i.e. aligned with scanner's *Z*‐axis, hereafter termed the axial direction). As a result, all BPET data acquired in this work was obtained with a 5 cm axial FOV. ROCSTAR also provides a MicroZed‐based Gigabit data link for transferring coincidence data from the scanner to the acquisition computer.^32^ All scanner DAQ components and power‐supplies are mounted on a mobile fan‐cooled electronics rack which is housed adjacent to the BPET–DBT gantry.

During data acquisition, the BPET scanner records raw signal waveforms for all coincidence events. This data is processed offline for position, energy, time‐of‐arrival calculations, and coincidence sorting, prior to generating list‐mode coincidence data. BPET detector and scanner calibrations were performed using an 18 mm thick ^18^F filled planar phantom that measured 48 cm (X) × 13 cm (*Z*). Image reconstruction was performed using an iterative list‐mode TOF algorithm^33^ with spherical image basis function,^34^ and incorporates all data corrections within the system model. Attenuation correction (described further in *section 2.3*) was performed using the DBT image to define the breast boundaries, and assuming uniform soft‐tissue composition. Random and scatter coincidences were estimated as a constant background derived from the tails of the prompt sinogram.

### 2.2 DBT – scanner design, data acquisition and image reconstruction

Conventional DBT utilizes a linear acquisition geometry where the x‐ray tube rotates around the patient's breast along a one‐dimensional arc in the mediolateral (ML) direction. Typically, 15 projections over a ±7.5° arc are acquired. This state‐of‐the‐art DBT scanner includes an additional posteroanterior tube motion and cranio‐caudal (CC) detector motion during DBT acquisition^35^ to improve overall spatial resolution and enhance image contrast.^36^ The scanner includes an XM‐1016T x‐ray tube (IAE, Italy), AXS‐2340 amorphous selenium detector with 85 µm detector element (Analogic, Canada), and a PMX x‐ray generator (Spellman, NY). Motion control is managed by a Parker‐Hannifin 6k8 controller, with tomosynthesis acquisition controlled via an Arduino MEGA‐2560 microcontroller. For all DBT acquisitions in this work, the x‐ray tube used a T‐geometry for x‐ray tube motion. Seven projections over ±7.5° in the ML direction were acquired, and an additional 8 (+14.3°) projections were acquired in the AP direction, with an acquisition time of ∼0.9 s per projection acquired using a step‐and‐shoot acquisition.

Images were reconstructed using commercial software (Briona v3.6, Real Time Tomography, PA), which automatically segments each DBT projection based on background signal (air) prior to filtered back‐projection. Native DBT images have an in‐plane resolution of 0.085  ×  0.085 mm^2^ and 0.5‐mm slice thickness.

### 2.3 BPET–DBT imaging – data acquisition, processing and corrections

**FIGURE 1:**
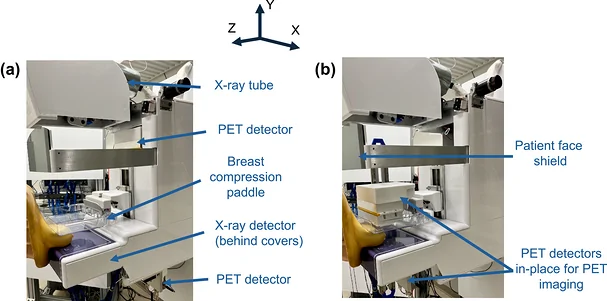
Photo of the BPET–DBT scanner. Shown are photos of the scanner acquiring (a) DBT, and (b) PET data using a deformable breast phantom to illustrate clinical imaging conditions. PET scanner is comprised of two detector heads, each measuring 20 cm (X) x 10 cm (Z), and utilizes 1.5 × 1.5 × 15 mm^3^ LYSO crystals. The DBT scan is sequentially followed by the PET scan with the breast compression paddle maintaining compression.

A single computer controls the motion of both the BPET and DBT systems and also performs DBT data acquisition, while a second computer controls BPET data acquisition. The gantry height is adjustable to facilitate breast positioning based on patient height. The DBT scan is performed first, using less breast compression than routine clinical imaging. Reduced compression is used because the subsequent BPET scan requires a longer acquisition time (nominally 5 mins) than DBT image acquisition, which is typically completed in under 10 s. The reduced compression improves patient comfort while maintaining adequate breast immobilization for BPET–DBT. Note that DBT image in this setup is non‐diagnostic and is used primarily for co‐registration and PET attenuation correction (AC). Hence the reduced breast compression is acceptable for this application. During DBT imaging, BPET detectors are parked at the back of the scanner (Figure 1a). After DBT, BPET acquisition begins with the breast remaining in the same compressed position. The x‐ray tube and detector retract as BPET detectors move forward and automatically center the breast between them (Figure 1b). As the DBT projections are computed without delay, they can also guide the axial positioning of the BPET detector heads.

While images acquired with the BPET–DBT scanner are intrinsically co‐registered, the spatial orientation between the two scanners needs to be mapped as a calibration step. This was achieved by imaging a single small diameter (<1 mm) ^18^F‐filled tube. This tubing was placed in a sine‐wave pattern extending through both the *X*‐*Y* and *X*‐*Z* planes, and over much of the BPET FOV to enable 3D co‐registration. A six‐degrees‐of‐freedom rigid‐body‐transformation aligns BPET and DBT images, storing the result as a co‐registration matrix which is subsequently applied to all DBT images.

Besides providing anatomic information, the DBT image is also used for PET AC. The DBT image is reconstructed with 1 × 1 × 1 mm^3^ voxels matched to the PET image voxel size, and the measured transformation matrix is used to co‐register the DBT and BPET images. The co‐registered DBT image is then used to delineate the compressed breast boundary and distinguish breast tissue from air, enabling generation of a patient‐specific attenuation map. For AC, the breast is assumed to have a uniform soft‐tissue composition within the segmented breast volume. Although volumetric breast density can be extracted from DBT images to derive patient‐specific attenuation factors,^37^ our previous work has demonstrated that assuming uniform soft‐tissue composition simplifies the AC process while maintaining PET accuracy with <5% bias.^38^

### 2.4 BPET performance characterization

#### 2.4.1 Spatial resolution

**FIGURE 2:**
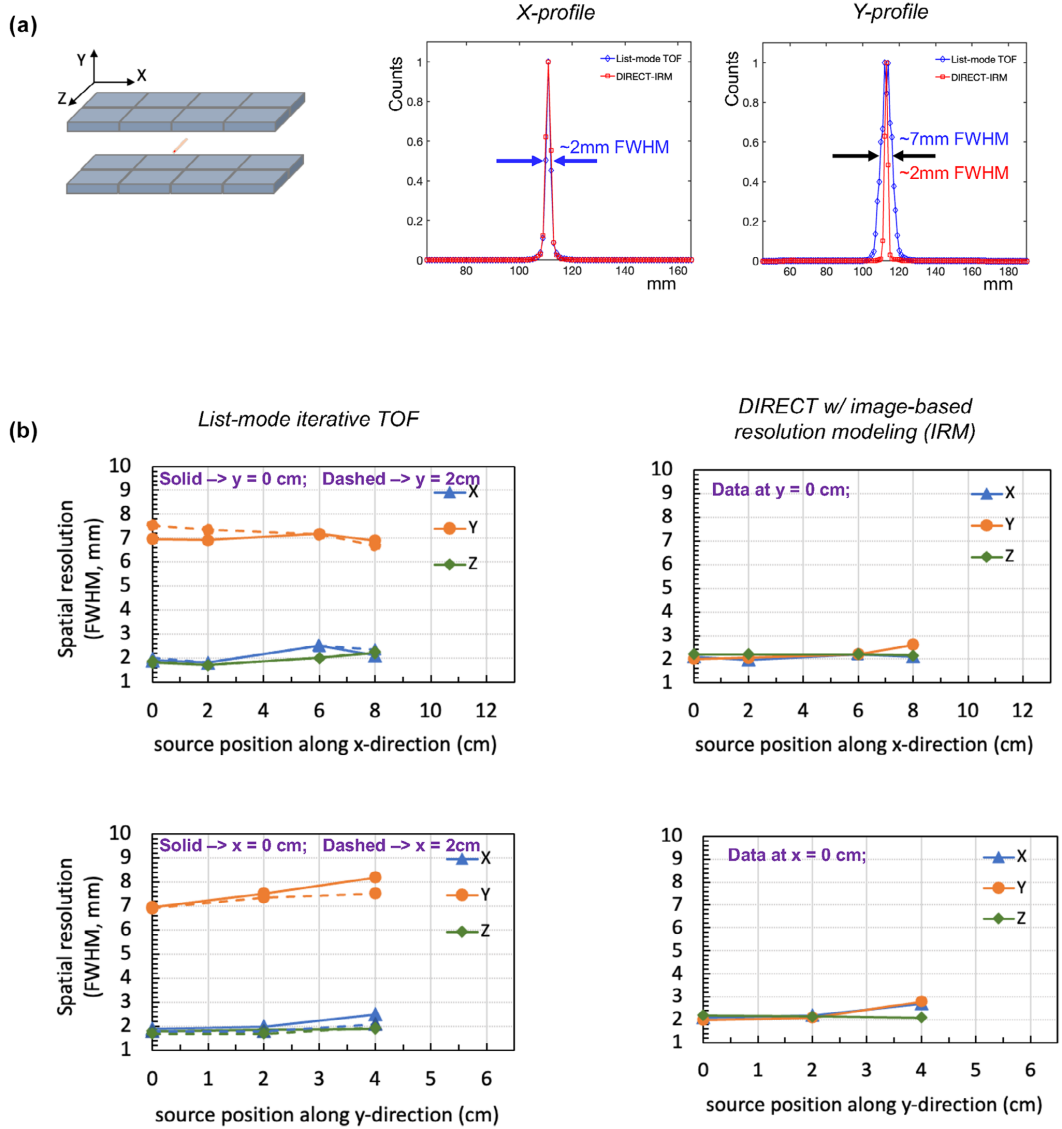
Scanner spatial resolution measured using an ^18^F filled capillary source – (a) schematic illustrating scanner orientation and source positioning. BPET data is acquired with the patient standing upright in front of the scanner and the breast under mild compression, positioned between the two detector panels illustrated in this *figure*. Image profiles along *X* and *Y* directions are also presented. (b) The source was translated at several locations along *X* and *Y* directions, and spatial resolution was measured using list‐mode TOF and DIRECT‐IRM reconstruction algorithms.

Spatial resolution was measured using a small capillary filled with ∼12 µCi of ^18^F placed near the BPET scanner's center (Figure 2a). The capillary source was also stepped along a few locations along the X (i.e. radial; at *y* = 0 and 2 cm) and Y (i.e. tangential; at *x* = 0 and 2 cm) directions, acquiring PET data for 5‐min at each location. Besides the iterative list‐mode TOF reconstruction algorithm, we used a previously developed spatially invariant, image‐based resolution model (DIRECT‐IRM),^39^ incorporated within the *Direct Image Reconstruction for TOF* reconstruction algorithm,^40^ which groups coincidence events directly into histo‐images. Data from point sources placed at *y* = 0 and *y* = ± 2 cm were averaged to generate this spatially invariant resolution model. The measurement at the center of the scanner was also used to characterize scanner energy and timing resolution.

#### 2.4.2 Scatter fraction and count rate performance

BPET count‐rate performance was assessed using a 3.2 mm diameter x 4.5 cm long plastic tube centered within a 20 cm (X) × 5 cm (Z) solid rectangular phantom composed of high‐density polyethylene. The phantom was 6.3 cm thick to represent a compressed breast. The tubing placed along the axial FOV (*Z*‐axis) of the scanner was filled with ∼100 µCi of ^18^F. The activity within the scanner FOV was chosen to reflect the expected upper level present during clinical imaging conditions, assuming an average breast volume of approximately 450 cc^41^ and reported radiotracer uptake of 0.07–0.2 µCi/cc for ^18^FDG and ^18^FES, which are commonly used in breast PET imaging. A 5‐min BPET acquisition was performed at multiple time points over several half‐lives of ^18^F radioactive decay. Count‐rate analysis was based on the NEMA NU‐4 methodology where the PET list‐mode data at each activity level were first binned into sinograms and the radial bins were re‐sampled into uniform bin spacing. For each projection angle, the radial bin containing the maximum count was identified, and the projections were shifted so that the peak positions were aligned. The aligned projections were summed to generate a single sinogram profile from which true (T), scatter (Sc), and random (R) coincidences were estimated. True coincidence events were estimated from counts within the central ±7 mm region of the profile. At low activity levels, scatter was estimated as the sum of the counts outside the ±7 mm region, and the scatter contribution within the ±7 mm peak region which was estimated as a linear interpolation of the background surrounding the peak region. Scatter fraction (SF) was defined as Sc/(T+Sc). At higher activity levels, analysis similar to that used earlier for estimation was performed to estimate the “background fraction,” where the background fraction is defined as (Sc+R)/(T+Sc+R). At low count‐rates (random coincidences <1% of true coincidences) the background fraction is the same as the scatter fraction while at higher count‐rates it is the fraction of total coincidence counts that are random and scatter coincidences. Based on the SF estimated from low activity level data, random coincidences can then be calculated from the background fraction. The noise equivalent count rate (NECR) was calculated as described by Strother et al.^42^

$$\mathrm{NECR}=\frac{T^{2}}{T+S_{c}+R}$$

where T, Sc, and R denote the true, scatter, and random coincidence rates, respectively. In addition, the total scanner coincidence count rate was measured at each time point to assess the count‐rate capability of the custom DAQ under clinically relevant conditions.

#### 2.4.3 Impact of random coincidences originating from activity present outside the FOV

The top BPET detector head does not incorporate end‐shielding in order to minimize the dead space between the patient's chest wall and the axial imaging FOV. However, 6 mm of lead shielding is used in front of the bottom detector head to reduce contributions from activity in the liver and bladder. We thus also assessed the effect of random coincidences from activity present outside the scanner FOV. Activity levels for this experiment were guided by our plans to use ^18^FES tracer in initial pilot human imaging studies. Unlike ^18^FDG, which exhibits diffuse uptake throughout the body, ^18^FES is predominantly metabolized by the liver, resulting in concentrated hepatic uptake.^43^ While this assumption is appropriate for ^18^FES, it overestimates the relative impact for typical ^18^FDG distribution. Breast activity was modeled using 25 µCi (∼0.14 µCi/cc) of ^18^F in a cylindrical phantom (5.1 cm in diameter and 8.6 cm long) positioned near the center of the scanner FOV. An additional ∼1200 cc ^18^F‐filled cylinder, representing the average combined liver and bladder volumes, was placed just outside the FOV to mimic a standing patient during BPET imaging. Data were acquired and BPET images were generated, with radial image profiles drawn through the images with up to 1 mCi (0.83 µCi/cc) present in the liver phantom (equivalent to 3.3 mCi ^18^FES injection^43^). The measured profiles were compared with those obtained in the absence of activity in the liver phantom.

#### 2.4.4 Micro‐deluxe Derenzo hot‐rod phantom imaging

The micro‐deluxe Derenzo hot‐rod phantom was imaged to assess overall image quality of the BPET scanner. The phantom was placed with the cylindrical axis aligned along the axial length (*Z*‐axis) of the scanner. A 60 min scan was acquired using the phantom filled with 30 µCi of ^18^F. In order to assess the scanner in‐plane (*X*‐*Z* plane) spatial resolution, the micro‐deluxe Derenzo hot rod phantom was also imaged by positioning it with its cylindrical axis aligned along the scanner's *Y*‐axis. Image reconstruction was performed using both reconstruction algorithms used to characterize spatial resolution (see prior Section 2.4.1).

#### 2.4.5 Quantitative imaging with a lesion phantom

A 5 cm diameter × 12 cm long cylindrical phantom with different sized spherical lesions was imaged to assess the BPET scanner's quantitative performance and its ability to resolve closely spaced lesions. Spherical lesions with diameters of 5, 8, 12, and 16 mm were individually positioned at the center of the phantom and filled to provide an 8:1 lesion‐to‐background uptake ratio. For each acquisition, the phantom was filled with ∼45 µCi (0.25 µCi/cc) of ^18^F, positioned near the center of the scanner, and imaged for 30 mins. To investigate the effect of scan duration on image quantification, the 30‐min list‐mode data was retroactively sub‐sampled and reconstructed to generate images corresponding to acquisition times of 2.5, 5, and 30 min. A single reconstruction was generated for the 30‐min acquisition, while 12 independent replicate 2.5 min reconstructions and 6 independent replicate 5 min reconstructions were generated from the list‐mode data for statistical analysis. While the 30‐min acquisition time is longer than our expected clinical imaging protocol, it permits evaluation of image quantification capability with minimal statistical noise contribution. Measurements with the 8‐mm diameter sphere were also repeated to assess the spatial dependence of image quantitation across the scanner FOV. For these measurements, the phantom was positioned at ±5 cm and ±8 cm along the *x*‐axis and +3 cm along the *y*‐axis, relative to the center of the scanner FOV (see Figure 5b for a scaled schematic of the sphere locations relative to the scanner FOV). To minimize variability in lesion contrast due to phantom filling, a single phantom fill was used for all measurements. The choice and number of measurement locations, while not exhaustive, were dictated by the need to select an adequate number of positions that could be evaluated using a single phantom fill while allowing evaluation of quantitation within the imaging FOV. The phantom was scanned for 15 min at the initial position (i.e. at the center of the FOV), and subsequent scan times at other locations were adjusted to account for radioactive decay, thereby maintaining equivalent count statistics across all acquisitions. For each acquisition, three independent replicate 5 min (or equivalent) reconstructions were generated.

For each image, the contrast recovery coefficient (CRC) was calculated using a NEMA‐style analysis with a spherical volume of interest (VOI) matched to the physical sphere diameter. The mean activity within the sphere (C_H_), and mean background activity (C_B_) measured by drawing a 10‐mm VOI in a uniform region of the phantom was used along with the assayed lesion‐to‐background uptake ratio (R_T_) to calculate CRC as

$$\text{CRC}=\frac{C_{H}/C_{B}-1}{R_{T}-1}$$

Subsequently, the ability to resolve closely spaced lesions was evaluated by placing two 8‐mm‐diameter lesions (1‐mm wall thickness) within the same 5 cm‐diameter cylinder. The two lesions with 15 mm wall‐to‐wall separation were filled to provide the same 8:1 lesion‐to‐background activity concentration ratio.

#### 2.4.6 Image uniformity

Image uniformity (background variability (BV), as defined by NEMA^27^) was assessed using a rectangular phantom with an active volume of 21 cm (*X*) × 5.5 cm (*Y*) × 6 cm (*Z*). The phantom had wall thickness of 1 cm along the lateral boundaries, and 5 mm along the *Y*‐axis, yielding an overall thickness representative of a breast compressed to 6.5 cm. The phantom was filled with 78 µCi (0.11 µCi/cc) of ^18^F, placed close to the center of the scanner, and imaged for 60 min. A 60 min scan was used to minimize the contribution of noise and isolate the effects of scanner geometry and image generation on image uniformity.

To assess the spatial dependence of image uniformity, BV was characterized for both the central‐FOV and edge‐FOV regions by placing forty‐five 1 cm diameter regions‐of‐interest (ROIs) distributed across three coronal (*X*–*Z* plane) image slices positioned at 0 cm and ±1.5 cm relative to the phantom center. Within each slice, fifteen VOIs were positioned near the edge of the FOV, fifteen were placed in the center, and fifteen were located midway between the edge and the center of the phantom. For each ROI, the mean image count was recorded. Image uniformity, quantified as the percent background variability (BV), was calculated as

$$\mathrm{BV}\;\left(\%\right)=100\;\times \;\frac{\sigma_{B}}{{\bar{C}}_{B}}$$

where ${\bar{C}}_{B}$ is the mean counts, and $\sigma_{B}$ is the corresponding standard deviation of the mean counts across all the ROIs in the region where BV was assessed. Within each slice, BV was calculated independently for the edge region (15 ROIs), central region (30 non‐edge ROIs), and overall slice (all 45 ROIs). In addition, a global BV was also calculated using all 135 ROIs across the three image slices.

The analysis was then repeated using 2 cm diameter ROIs. The inclusion of both these ROI sizes ensures that uniformity is evaluated over the representative volumes typically encountered in breast imaging, While the 1 cm diameter is representative of small lesions, the 2 cm is representative of larger tissue regions and typically provides a more stable estimate of background uniformity.

### 2.5 BPET–DBT phantom imaging

#### 2.5.1 Co‐registration accuracy with custom lesion phantom

BPET–DBT imaging was assessed with a custom 3D‐printed phantom built using Polyethylene Terephthalate (with Glycol), a popular material which provides a smooth surface finish during 3D printing. The body of the phantom was largely hollow but included two carefully placed cavities for holding a 1 cm diameter (1 mm wall thickness, 3 cm center‐to‐center spacing) ^18^F‐filled sphere in each cavity. The 6.8 cm‐thick phantom had a D‐shaped outline, designed to mimic a compressed breast as seen during conventional DBT imaging. The two spheres were filled with 24 µCi of activity and placed near the center of the BPET scanner. A DBT scan was followed by a 10 min BPET scan. In addition to visually assessing the quality of the co‐registered BPET–DBT images, the accuracy of co‐registration was quantified by calculating the relative volumetric displacement of the sphere centers between the BPET and DBT images.

#### 2.5.2 Quantitative accuracy with NEMA NU‐4 image quality phantom

We imaged the NEMA NU‐4 Image Quality phantom ^28^ which is commonly used to assess image quality and accuracy of PET data corrections in small‐animal PET scanners. The phantom was positioned with its cylindrical axis aligned along the scanner *z*‐axis. Because the contrast recovery coefficient measurements specified by the NU‐4 Image Quality phantom are not representative of breast imaging performance, the analysis focused on image uniformity and the accuracy of PET data corrections. The phantom was filled with 27 µCi of ^18^F, and a 20 min BPET scan was acquired following DBT acquisition. BPET images were reconstructed using the iterative list‐mode TOF reconstruction algorithm. PET data corrections were performed using both the DBT‐derived attenuation map and an analytical attenuation model of the phantom,^44^ and the two images were compared to assess the accuracy of the DBT‐derived attenuation map. As prescribed by the NU‐4 standard, image uniformity and accuracy of data corrections were evaluated over the different sections of the reconstructed image of the NU‐4 image quality phantom. Image uniformity was measured using a 22.5 mm diameter × 10 mm long VOI covering the central 75% of the uniform section. The accuracy of PET data corrections was assessed using a centrally positioned 4 mm diameter × 7.5 mm long VOI, corresponding to half the diameter of the air‐ and water‐filled compartments.

### 2.6 Pilot BPET–DBT human study

Pilot human imaging using BPET–DBT was performed under an IRB approved protocol that utilized ^18^FES radiotracer in subjects with newly diagnosed ER+ breast cancer. After providing informed consent, the subject was injected with 167 MBq (4.5 mCi) ^18^FES and imaged on the BPET–DBT scanner, 95 mins post‐injection, in a standing position. The BPET–DBT scan was performed under mild breast compression (7.8 cm compressed breast thickness). The DBT scan used patient‐specific kV‐mAs (35 kV, 75 mAs), as per commercial manufacturers. Only CC views were acquired for this pilot investigation, as the BPET–DBT gantry rotation mechanism necessary to obtain mediolateral‐oblique views was not implemented. A 5 min PET scan was performed for each breast (i.e. with both breasts imaged sequentially) following the DBT scan.

For comparison to WB‐PET, the BPET–DBT scan was preceded by a WB‐PET scan (55 mins post‐injection) on the PennPET Explorer,^45^ a total‐body PET scanner with sensitivity higher than clinical WB‐PET scanners, per a separate study protocol and consent. ^18^FES has been shown to accumulate within ER‐expressing tissue in 20–30 mins and achieve biological equilibrium within 60 mins after injection.^43^, ^46^ As both of these PET scans were acquired within the steady‐state period, no change is ^18^FES uptake is expected between BPET and PennPET acquisitions. A 4‐min single bed position WB‐PET scan was acquired, and images were generated with 2 × 2 × 2 mm^3^ voxels, using an iterative list‐mode TOF algorithm.^33^ The co‐registered BPET–DBT images were compared as were the BPET and PennPET images. Besides visual correlation, mean standardized uptake value (SUV_mean_) and tumor‐to‐background levels were also compared between the PET images. A 2.4 cm × 1.3 cm tumor region was identified on the BPET image based on the extent of radiotracer uptake visible within the BPET image.  Ten non‐overlapping 5‐mm diameter ROIs were placed, and SUV_mean_ was calculated as the average of the mean SUV values measured in these ROIs. Multiple small ROIs were used instead of a single tumor ROI to provide consistent sampling of the tumor across BPET and PennPET images, and to minimize potential bias in SUV measurements arising from inclusion of the necrotic (low uptake) core at the center of the tumor.

## 3 RESULTS

### 3.1 BPET performance characterization

#### 3.1.1 Spatial resolution

With the source near the scanner's center, Figure 2a shows image profiles drawn along the *X* and *Y* directions. The choice of iterations for both reconstruction algorithms – 5 for list‐mode TOF and 50 for DIRECT‐IRM, was based on measured phantom data acquired with spheres in a warm background showing signal convergence and is consistent with our experience using other clinical scanners.^45^ The higher number of iterations for DIRECT‐IRM is consistent with the inclusion of resolution modeling, which generally leads to slower convergence.^39^ This iteration choice has been consistently applied throughout this work.

Resolution of 2 mm FWHM along the *X* and *Z* directions, and *Y* resolution of ∼7 mm FWHM was measured. Spatial resolution was also characterized for a few source locations along the *X* and *Y* direction and showed minimal deterioration (Figure 2b). DIRECT‐IRM mitigated resolution loss along *Y*‐direction, which is caused by the scanner's dual‐panel design and parallax error. System timing resolution of 425 ps FWHM and energy resolution of 16% FWHM was measured.

#### 3.1.2 Scatter fraction and count rate performance

**FIGURE 3:**
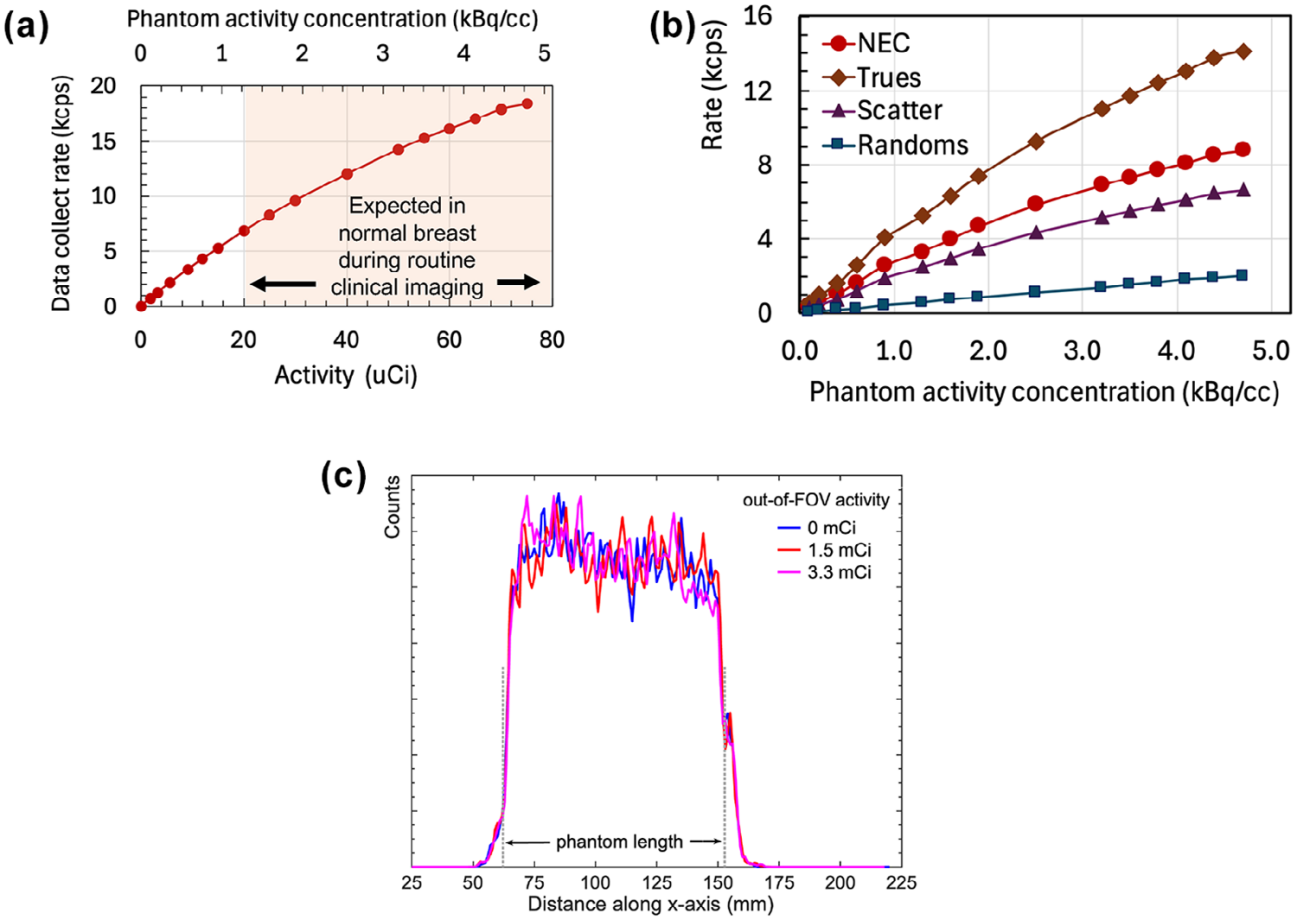
(a) BPET scanner count rate performance evaluated using a ^18^F‐filled line source placed within a solid rectangular polyethylene phantom. (b) Noise equivalent count rate (NEC), True, Scatter and Randoms count rates as a function of phantom activity concentration. (c) Image profiles drawn from imaging an ^18^F‐filled uniform cylinder placed within the scanner FOV and an additional cylinder containing varying activity levels to assess the impact of random coincidences from out‐of‐field‐of‐view activity. Note that the slightly elongated tails beyond the phantom length arise from activity in the phantom end screw and fill holes.

Figure 3a shows the count rate performance for the BPET scanner. The scanner prompt collect rates are fairly linear over the entire activity range. Measurements demonstrate BPET scanner has sufficient throughput to support clinical use of this scanner. A scatter fraction of 32% was also measured with this line source phantom at the lowest activity level. For acquisitions with higher activity within the FOV, minimal additional tails were observed in the prompt sinograms, indicating minimal contribution from random coincidences at these activity levels which are also representative of our patient imaging protocol. Figure 3b shows the noise equivalent count rate (NECR), True, Scatter, and Randoms count rates. NEC has a peak value of 8.8 kcps at an activity concentration of 4.7 kBq/cc.

#### 3.1.3 Impact of random coincidences originating from activity present outside the FOV

When evaluating the effect of random coincidences arising from activity outside the FOV (Figure 3c), we did not observe any additional tails in the image profiles that would typically be associated with the presence of random coincidences.

#### 3.1.4 Micro‐deluxe Derenzo hot‐rod phantom imaging

**FIGURE 4:**
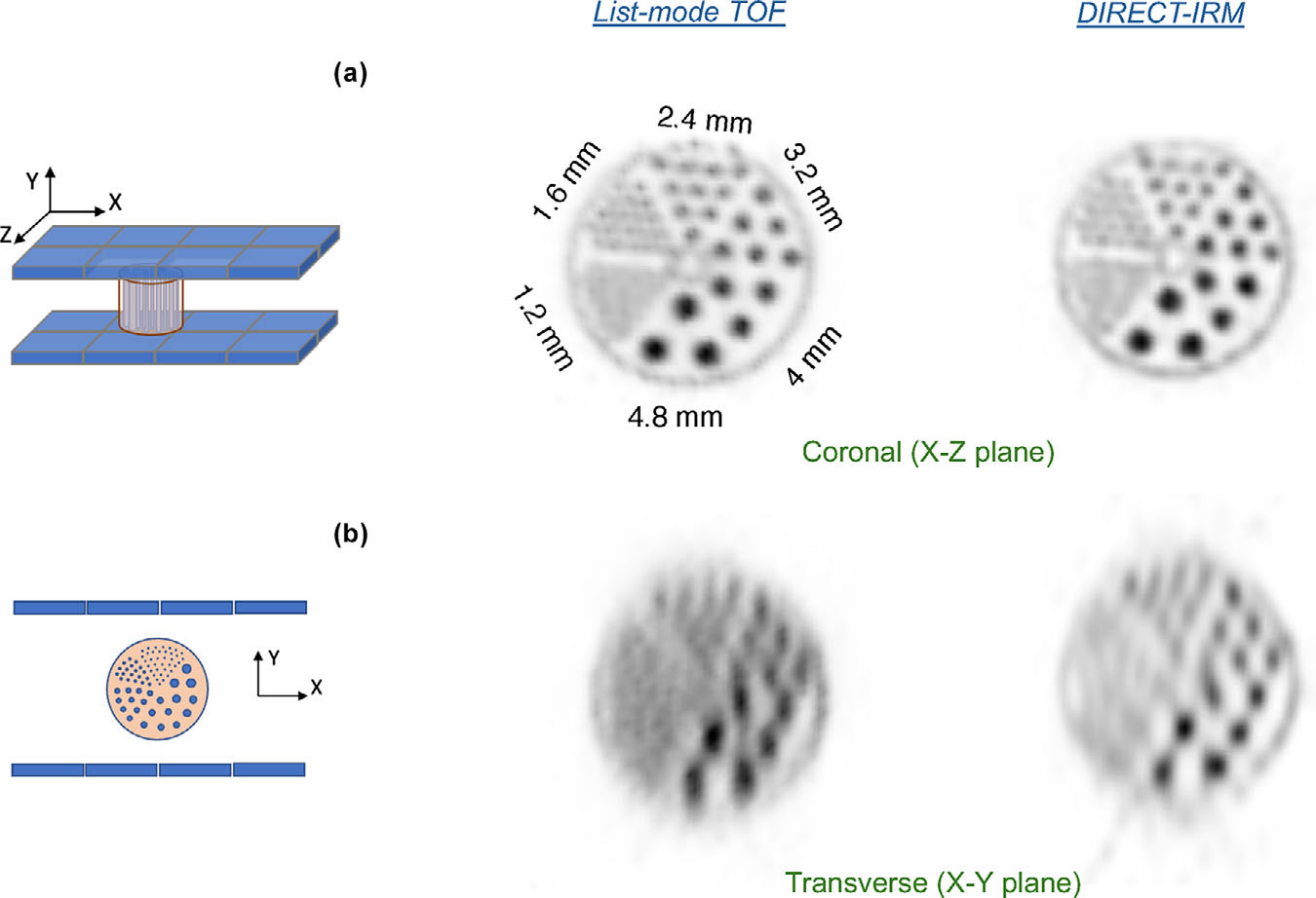
(a) Central coronal image slice, and (b) central transverse image slice from imaging the micro‐deluxe hot rod phantom, reconstructed using the two image reconstruction algorithms.

Figure 4a presents the central coronal slice from imaging the micro‐deluxe Derenzo hot rod phantom with its cylindrical axis aligned along the scanner's *Y*‐axis. Images reconstructed using list‐mode TOF (*left*), and DIRECT‐IRM (*right*) algorithms are shown. Both images show excellent discrimination of the 2.4 mm rods, and good discrimination of the 1.6 mm rods. Figure 4b shows central transverse slice from the same phantom imaged with its cylindrical axis aligned along the axial length of the scanner. Improved discrimination for the 2.4 mm rods is observed in the transverse (*X*‐*Y*) image plane with DIRECT‐IRM.

#### 3.1.5 Quantitative imaging with a lesion phantom

**FIGURE 5:**
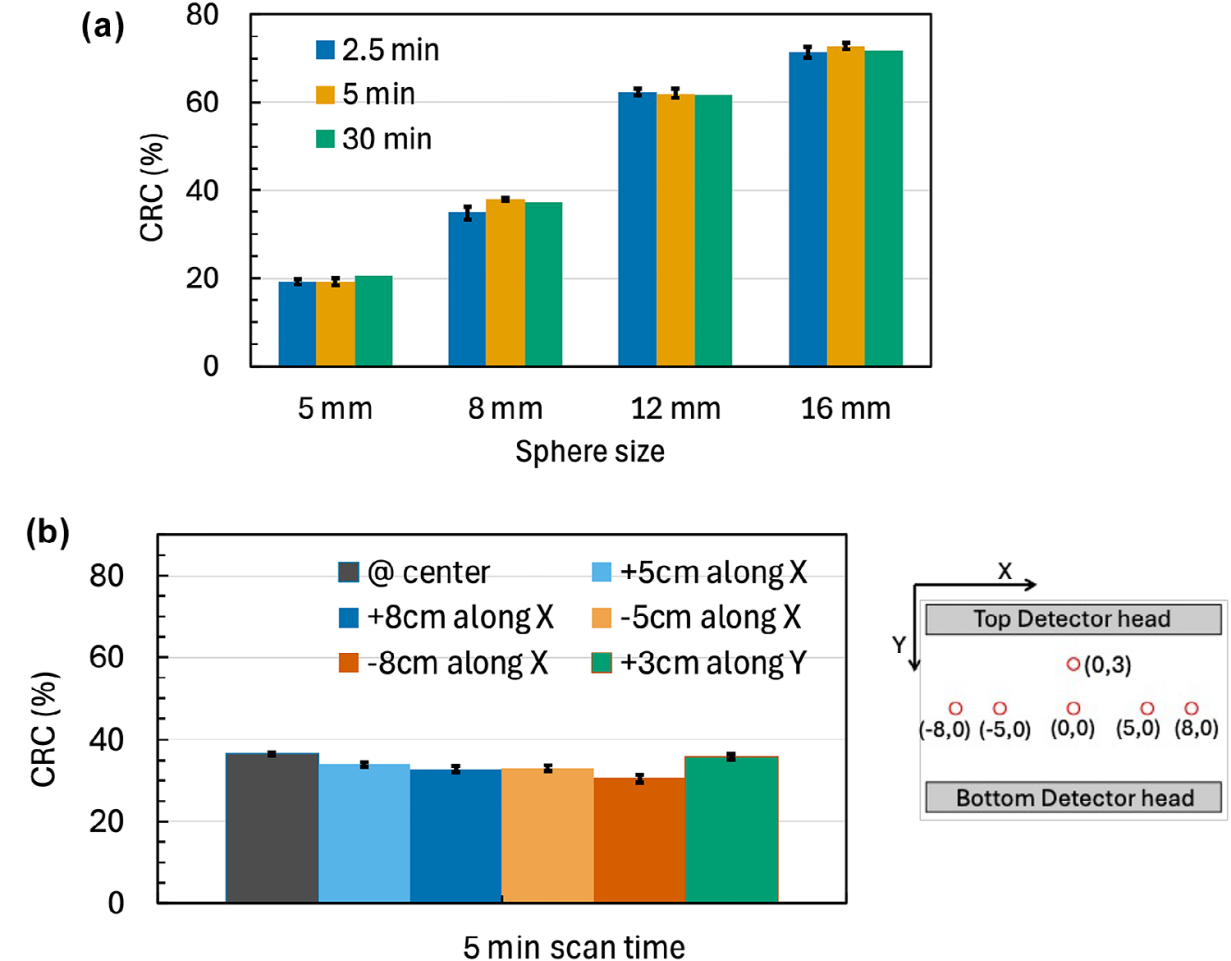
Contrast recovery coefficient (CRC) measured using (a) spheres of varying diameters, and (b) an 8‐mm diameter sphere placed at different positions within the scanner FOV. A scaled schematic of the sphere locations relative to the scanner FOV, corresponding to the measurement positions in (b), is shown on its right. For all measurements sphere was placed in a 5 cm diameter × 12 cm long cylindrical phantom with 8:1 activity uptake ratio.

**FIGURE 6:**
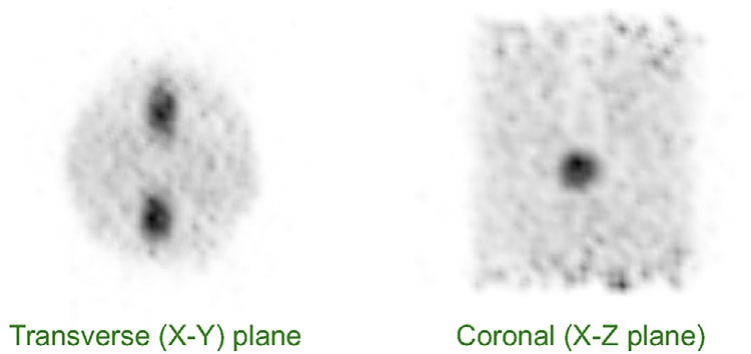
Central transverse and coronal image slice of the cylindrical phantom with two 8 mm diameter spherical lesions with 15 mm wall‐to‐wall separation placed in a warm background with 8:1 uptake.

Lesion quantification measurements (Figure 5) and reconstructed images (Figure 6) demonstrate that the BPET scanner achieves good image quality and provides reliable quantitative performance with a 5 min acquisition time. As expected, CRC increased with sphere size across all scan time durations (Figure 5a), reaching 19%, 38%, 62% and 73% for the 5, 8, 12, and 16 mm diameter spheres respectively. For each sphere size, as expected, CRC also remained relatively stable across acquisition times ranging from 2.5 to 30 min. Measurements using the 8 mm sphere individually positioned at multiple locations within the scanner FOV demonstrate that CRC was relatively constant across the different sphere positions (Figure 5b). For the 5‐min acquisition, the highest CRC (37%) was measured at the center of the scanner, whereas the lowest CRC (31%) was observed at the farthest off‐center position measurement located at −8 cm along the *x*‐axis.

Transverse and coronal images of the phantom containing two 8 mm lesions separated by 15 mm (Figure 6) demonstrate the scanner's ability to clearly resolve the 8 mm diameter lesions placed in a warm background.

#### 3.1.6 Image uniformity

**FIGURE 7:**
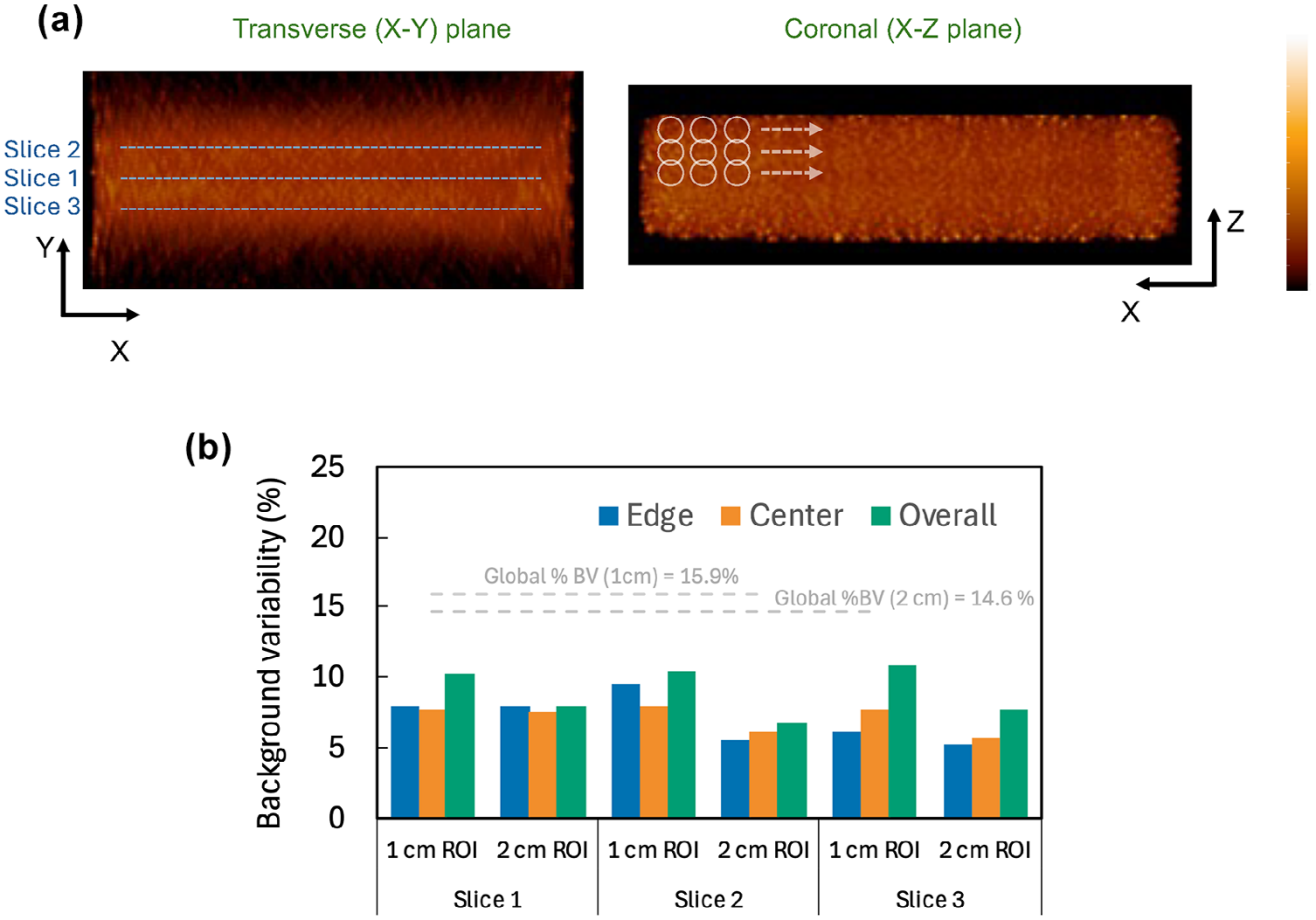
Central transverse and coronal image slices of a rectangular phantom used to assess BPET image uniformity. The phantom was uniformly filled with 0.11 µCi/cc of ^18^F and positioned near the center of the scanner. The three *cyan lines* on the transverse image slice indicate the locations of the three coronal slices used for BV analysis (0 and ±1.5 cm relative to the phantom center), while the *white circles* on the coronal image slice indicate locations of the forty‐five 1 cm‐ and 2 cm‐diameter circular regions of interest within each coronal image slice. Fifteen VOIs were placed along each of the three axial (*Z*) locations indicated by the *white circles*. (B) Background variability (BV), calculated from the 1 and 2 cm diameter ROI measurements in each slice, is shown for the edge, central, and overall regions. Results for global BV (calculated over all ROIs distributed over the three slices) are shown by the dashed grey lines.

Central image slices of the rectangular uniform phantom, and image uniformity, quantified by the NEMA image BV, are summarized in Figure 7. For the 1 cm diameter ROIs, BV for the central and edge regions ranged from approximately 6%–9.5%, while the overall BV within each of the three slices ranged from 10%–11%. With the larger 2 cm diameter ROIs, the BV was reduced: 5%–8% in the central and edge regions and 7%–8% overall for the three slices. Global BV was higher with values of 15.9% and 14.6% for the 1 and 2 cm diameter ROIs, respectively.

### 3.2 BPET–DBT phantom imaging

#### 3.2.1 Co‐registration accuracy with custom lesion phantom

**FIGURE 8:**
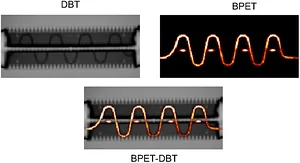
Shown above (*top*) are central coronal (*X*–*Z* image plane) DBT and BPET image slices from imaging a single <1 mm diameter ^18^F‐filled tube used to perform BPET‐DBT co‐registration calibration. The tube was arranged in a sine‐wave pattern that extended along both *X*–*Z* and *X*–*Y* planes. In the BPET image the short bright segments observed between portions of the sine‐wave pattern correspond to connecting sections of the tube arranged along the *X*‐*Y* plane. The co‐registration matrix generated from BPET‐DBT co‐registration (*bottom*) is used as a calibration reference for automatic co‐registration of all future BPET‐DBT imaging.

**FIGURE 9:**
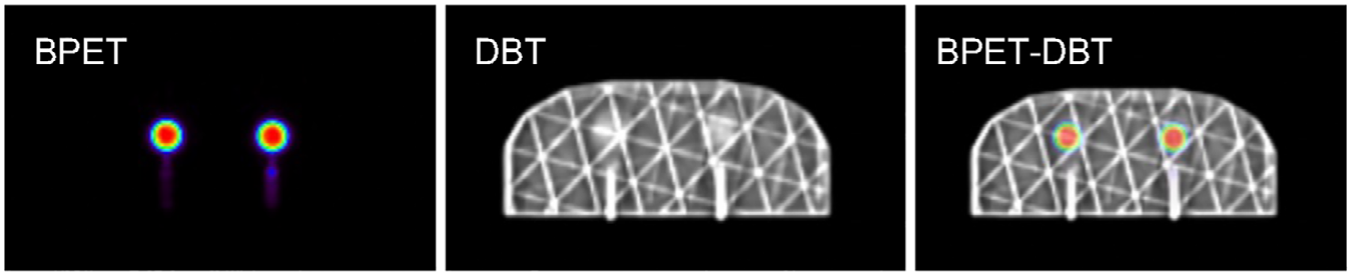
Co‐registered central coronal image slices from BPET–DBT imaging of a custom 3D‐printed phantom having two 1 cm diameter ^18^F‐filled spheres placed within a D‐shaped phantom. Shown are BPET, DBT, and co‐registered BPET–DBT image slices. Note the hatched pattern in the DBT image is from 3D printing of the phantom.

Figure 8 shows the central coronal slices from the BPET, DBT, and co‐registered BPET–DBT images of the <1 mm diameter ^18^F‐filled tube used to determine the transformation matrix required for co‐registration of subsequent BPET–DBT scans. Figure 9 shows the central coronal slice from BPET (left), DBT (middle) and co‐registered BPET–DBT image (right) from scanning the custom BPET–DBT phantom. Besides the excellent visual correlation between the BPET and DBT images, an average volumetric (3‐D) co‐registration accuracy of 0.8 mm (0.7 mm in the 2‐D *X*–*Z* image plane) was measured for the two spheres placed within this phantom.

#### 3.2.2 Quantitative accuracy with NEMA NU‐4 image quality phantom

**FIGURE 10:**
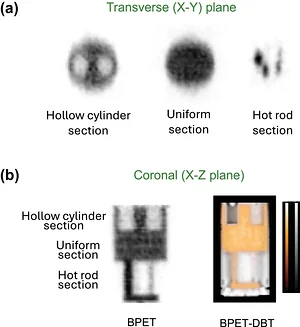
Reconstructed images of the NEMA NU‐4 Image Quality phantom acquired with the BPET–DBT scanner using DBT‐based PET attenuation correction. (a) Central transverse (*X–Y*) BPET image slice showing the hollow‐cylinder, uniform, and hot‐rod sections of the phantom. (b) Central coronal (*X–Z*) BPET image (*left*) and the co‐registered BPET–DBT image (*right*) demonstrating spatial alignment between the BPET and DBT datasets.

Figure 10 summarizes the BPET images of the NEMA NU‐4 Image quality phantom reconstructed using the DBT‐derived attenuation map for PET data corrections. The transverse BPET images demonstrate good uniformity within the uniform region, clear delineation of the hollow cylinders, and visualization of rods ≥2 mm in diameter in the hot‐rod section. The coronal BPET and co‐registered BPET‐DBT images demonstrate accurate image co‐registration between the BPET and DBT datasets. Image uniformity was 8.8%, while spill‐over ratios of 20% and 24% were measured for the air and water compartments, respectively. No appreciable differences were observed between BPET images reconstructed using DBT‐derived and analytical attenuation map.

### 3.3 Pilot BPET–DBT human study

**FIGURE 11:**
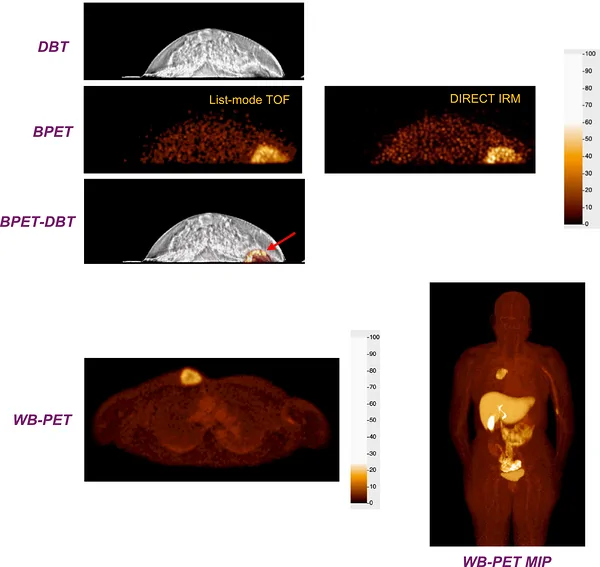
Single central coronal (*x*‐*z* plane) slice of DBT, list‐mode TOF and DIRECT‐IRM reconstructed BPET, and co‐registered BPET–DBT images from the right breast of the subject are show on the *top*. There was no lesion in the left breast. Also included for comparison in the *bottom* are the transverse slice and MIP image from imaging the patient on the PennPET Explorer total‐body PET scanner.

Figure 11 shows central coronal slice of the DBT (*top*), BPET reconstructions from both list‐mode TOF and DIRECT‐IRM reconstruction algorithms (*top, middle*), and co‐registered list‐mode TOF BPET–DBT (*top, bottom*) images from the patient's right breast (side of malignancy). The left breast did not have a lesion. There was excellent correlation between the DBT scan and the focal ^18^FES uptake observed on both BPET images. BPET images reconstructed using both the reconstruction algorithms and the PennPET image demonstrated similar tumor‐to‐background ratio and also consistent visualization of the necrotic tumor core (*red arrow* in the BPET–DBT image in Figure 11). Focal uptake comparison measurements demonstrated an average SUV_mean_ of 9.7 ± 1.5 for BPET and 8.2 ± 1.6 for PennPET. Because the BPET image was acquired with the patient in an upright position and the breast in mild compression, precise anatomical correlation between the two images is challenging, and limited a more detailed analysis between the two PET scans. Nevertheless, quantitative assessment of the lesion, as described above, indicated that the BPET image is comparable to the whole‐body PET image.

## 4 DISCUSSION

This paper presents the design, physical characterization, and imaging performance of a dedicated BPET scanner. The primary motivation for using a dual‐panel scanner design is its seamless integration into current clinical workflow, particularly within a single gantry alongside tomosynthesis/mammography systems that are routinely used in clinical imaging. Besides adding practicality, it also offers the additional benefits from a dedicated scanner, such as higher spatial resolution and reduced cost.

Technical contributions in several areas were necessary for the development of this scanner—development of a high spatial resolution and time‐of‐flight PET detector, custom time‐of‐flight data acquisition electronics, data calibrations and image reconstruction—all of which are relevant to maintaining the quantitative accuracy in a PET scanner having limited angular coverage. The BPET scanner employs a PMT‐based detector, as the multi‐anode PMT met our design requirements, and electronics for PMT readout were under development concurrently. Advancements in TOF resolution achievable with current SiPM‐based detectors would further enhance the imaging capabilities of the BPET–DBT scanner. An earlier conference proceeding ^47^ briefly described a preliminary implementation of the BPET–DBT hardware while detector electronics and timing calibrations were still under development. In contrast, the present work reports on the fully developed, calibrated, and validated system, including comprehensive imaging performance characterization and improved TOF resolution (425 ps versus approximately 700 ps in the preliminary work), results that were not presented in the earlier conference publication.

Spatial resolution measurements on the BPET scanner demonstrate an in‐plane (*X*–*Z*) resolution of 2 mm. Although analytical reconstruction is typically used to assess spatial resolution, it was not feasible for this dual‐panel scanner design with incomplete angular coverage. Therefore, iterative reconstruction algorithms, with an iteration number based on contrast convergence during reconstruction of spherical lesions placed within a warm background was utilized. These reconstruction parameters were consistently applied to both phantom and clinical imaging studies. Beyond the effects of partial angular coverage, the reduced separation between detector panels in comparison with WB‐PET, exacerbates parallax effects and also contributes towards spatial resolution degradation. Our results (Figure 2) also show that image‐based resolution modeling (DIRECT‐IRM) helps mitigate the overall resolution loss along the y‐direction. While our initial experimental evaluation of DIRECT‐IRM is promising, a previous simulation study^39^ demonstrated that a spatially variant IRM achieves more accurate and uniform lesion uptake quantification across the FOV. Further work is ongoing to practically implement a spatially variant resolution model to better characterize the system behavior using only a modest number of point‐sources for calibration.^48^ Consequently, in this work, we have not performed a detailed comparison of the advantages of using DIRECT‐IRM in the pilot study.

Count‐rate measurements spanned the typical ^18^F activity range expected within the scanner FOV during routine clinical imaging. The measurements demonstrate that, with the use of a high‐speed waveform sampling DAQ, BPET scanner allows data collection with minimal dead‐time and provides sufficient throughput to support the intended clinical application. The NECR curve reached a maximum value of 8.8 kcps at an activity concentration of approximately 4.7 kBq/cc (Figure 3b). Count‐rate performance evaluations of dedicated breast PET scanners utilizing limited‐angle geometry have primarily focused on characterizing the system throughput, thus limiting direct comparison with the peak NECR reported in this work.^18^ However, the peak NECR is lower in comparison with full‐ring dedicated breast PET scanners,^15^, ^19^, ^21^ primarily because of the reduced coincidence sensitivity associated with incomplete angular coverage in a limited‐angle scanner geometry. More importantly, the count‐rate measurements demonstrate that DAQ provides sufficient throughput over the clinically relevant activity range to support clinical use. Higher activity levels were not investigated because of a manufacturing issue affecting two PMTs that caused high‐voltage sagging at elevated count rates, limiting the activity range in this study to below those at which this behavior occurred. This issue was confined to two detector modules and thus is not considered a fundamental limitation of the BPET scanner design. Importantly, the present measurements demonstrate that the scanner provides sufficient count‐rate performance within the expected clinical operating range.

Unlike WB‐PET, where the scatter contributions are influenced by the large imaging volume and surrounding anatomy, scatter in dedicated breast PET is generally lower due to the smaller imaging volume and is primarily determined by the breast size and scanner energy resolution. With system‐wide energy resolution of 16% and a lower energy threshold of 410 keV, a scatter fraction of 32% was measured for activity within the FOV. This value is consistent with previously reported scatter fractions for dedicated breast PET scanners where values in the 20%–35% have been reported.^11^, ^19^, ^49^, ^50^ The phantom used in this study was designed to approximate a large, compressed breast geometry (20 cm wide × 6.3 cm thick). Therefore, the measured scatter fraction likely provides a conservative estimate of scatter contribution expected during routine breast imaging. These results thus indicate that scatter contributions would not be a limiting factor for the proposed clinical evaluation. Although scatter and random coincidences were estimated as a constant background in this work, established scatter and randoms correction techniques that are routinely used in clinical PET imaging will be implemented in future studies. Specifically, scatter correction will use the single scatter simulation approach,^51^, ^52^, ^53^ and randoms coincidences will be estimated using the delayed‐window coincidence technique.^54^ While the firmware required for delayed‐window processing is still under development, BPET DAQ has been designed to support this functionality. In the meantime, the combination of good system‐wide energy resolution and narrow hardware acquisition coincidence window (±2.2 ns) effectively reduces the random coincidences within the activity range relevant for clinical imaging. Additionally, measurements with activity positioned outside the FOV demonstrated no observable increase in random‐related tails in the reconstructed profiles (Figure 3c), suggesting that out‐of‐FOV activity expected during clinical imaging does not substantially degrade quantitative performance.

With system timing resolution of 425 ps, the DAQ also provides good TOF imaging capability necessary for realizing adequate image quality in a scanner with limited angular coverage. Supporting these spatial resolution measurements, imaging of the micro‐deluxe Derenzo hot rod phantom shows excellent in‐plane (*X*‐*Z*) discrimination of the 2.4 mm and 1.6 mm rods, and good discrimination of 2.4 mm diameter rods in the out‐of‐plane (*X*‐*Y*) direction after IRM (Figure 4).

Lesion phantom imaging demonstrated that the BPET scanner provides good image quality and quantitative performance using a 5 min acquisition, which represents the planned clinical acquisition time for imaging a single breast. As expected, CRC increased with lesion size due to reduced partial volume effect, achieving reliable contrast recovery for sub‐centimeter lesions, with CRC values of 38% and 62% for the 8 and 12 mm spheres, respectively (Figure 5a). Although direct comparison of CRC with other dedicated breast PET scanners employing limited‐angle geometries is challenging because of differences in methodology and lesion‐to‐background ratio, the measured CRC values, calculated using the mean signal within the VOI, suggest higher contrast recovery than that reported for the PEM Flex Solo II.^18^ While several factors, including image reconstruction, may contribute to the observed improvement, the BPET scanner's TOF resolution of 425 ps is consistent with the expected benefits of TOF in limited‐angle PET.^25^, ^26^ Specifically, TOF has been shown to mitigate some of the effects of incomplete angular sampling, resulting in improved image quality and more accurate quantification of lesion uptake. The reported CRC is also comparable to that reported for several other full‐ring dedicated breast PET scanners,^55^, ^56^ as well as the PennPET total‐body PET scanner,^57^ which reported a CRC of approximately 60% for the 13 mm sphere. These results thus suggest that the BPET scanner can provide useful quantitative assessment of clinically relevant sub‐centimeter breast lesions while retaining the advantage of useful clinical workflow with co‐registered DBT imaging.

CRC values also remained relatively stable across acquisition times ranging from 2.5 to 30 min for all sphere sizes, indicating that lesion quantification is robust and that extending the scan duration beyond 5 min provides limited improvement in quantitative accuracy. This also suggests that quantitative performance for BPET is predominantly limited by the reduced out‐of‐plane spatial resolution (y‐direction) associated with the limited‐angle acquisition geometry of the BPET scanner.

Measurements with the 8 mm diameter lesion phantom acquired at multiple locations within the scanner FOV demonstrated only modest variations in CRC (Figure 5b). Characterizing the uniformity of CRC over the imaging FOV is particularly important in a limited‐angle PET scanner because incomplete angular sampling can produce position‐dependent quantitative bias across the imaging volume. Compared with previous evaluations of limited‐angle breast PET systems, such as the Naviscan PEM Flex Solo II,^18^ which reported higher location‐dependent variations in quantitative recovery attributable to the acquisition geometry, the present initial valuation demonstrates relatively smaller CRC variation among the sampled FOV locations, suggesting improved quantitative uniformity within the imaging FOV. The incorporation of a spatially variant IRM is expected to improve CRC for all sphere sizes while also further improving its spatial uniformity across the FOV, thereby enhancing quantitative accuracy and consistency throughout the imaging volume. Furthermore, reconstructed images of two 8‐mm lesions separated by 15 mm clearly resolved both lesions within a warm background (Figure 6), demonstrating that the system provides sufficient spatial resolution and contrast to resolve closely spaced sub‐centimeter lesions.

Image uniformity of 8.8% was measured using the NEMA NU‐4 Image quality phantom. This value is slightly higher than the 4.5%–5.8% image uniformity reported for some full‐ring dedicated breast PET scanners,^15^, ^21^ and the difference likely reflects the limited‐angle scanner geometry employed by BPET which is expected to contribute towards some of the non‐uniformity. However, as the NEMA NU‐4 test was developed for small animal PET scanners, its relatively small uniform region does not fully capture the spatial image uniformity of a limited‐angle PET system. To better assess uniformity across the imaging volume, additional measurements were therefore performed using a larger rectangular uniform phantom (Figure 7). Both 1 and 2 cm diameter ROIs were used in order to assess the robustness of quantitative measurements across clinically relevant ROI sizes. BV generally decreased across all regions when the 2 cm ROI was used. Global BV calculated over three coronal slices was higher than overall BV within each slice, indicating some variability across the coronal slices likely due to limited angular coverage of the system. Although a direct comparison with other PET scanners is complicated by differences in phantom designs, acquisition and reconstruction protocols, and experimental methodology, the measured BV values fall within the range reported for other limited‐angle dedicated breast PET systems.^18^ In comparison, full‐ring dedicated breast PET scanners have reported BV values in the range of approximately 6%–12%,^19^ while total‐body PET scanners have achieved BV values below 10% for acquisition times as short as 3 min.^57^, ^58^ The lower BV values are consistent with the higher sensitivity and complete angular sampling provided by these systems. Although the BPET design results in slightly higher BV, it also provides an open configuration that enables seamless integration with DBT and routine clinical breast imaging workflows. As BPET is intended to support applications beyond cancer detection, the trade‐off of slightly higher BV might be acceptable, and should be considered in the context of the scanner's overall imaging performance.

When imaging the custom BPET–DBT phantom, both scanners clearly visualized the two 1 cm spherical lesions, demonstrating excellent agreement between the intrinsically co‐registered BPET–DBT images (Figure 9)*. F*urther, the excellent spatial alignment confirms accurate co‐registration calibration and demonstrates the ability to acquire intrinsically co‐registered functional and anatomical images.

In addition to image uniformity, the NEMA NU‐4 Image Quality phantom was also used to evaluate the quantitative accuracy of the proposed DBT‐derived attenuation correction. BPET images reconstructed using the DBT‐derived attenuation map demonstrated good delineation of the phantom structures and image quality (Figure 10). The close agreement with images reconstructed using the analytical attenuation map indicates that the DBT‐derived attenuation map provides quantitative performance comparable to the analytical attenuation model.

Spill‐over‐ratio of 20% and 24% were measured for the air‐ and water‐filled compartments, respectively. These values were slightly higher than those reported for full‐ring dedicated breast PET scanners, where spill‐over ratios of approximately 2%–20% have been reported depending on the reconstruction protocol.^15^, ^21^ However, they are substantially lower than the approximately 40%–60% reported by Luo et al.,^59^ for another limited‐angle breast PET system. In a full‐ring PET scanners with high and isotropic spatial resolution, low spill‐over ratios are closely associated with accurate PET data corrections. However, in limited‐angle PET systems, spill‐over is also influenced by the spatial resolution degradation related to the incomplete angular sampling. The similar spill‐over ratios obtained using the DBT‐derived and analytical attenuation maps further suggest that the slightly increased spill‐over is likely not due to inaccuracies in the DBT‐based attenuation correction.

Our first human imaging study using the ^18^FES tracer in an ER+ patient demonstrates excellent correlation with DBT, and also the companion WB‐PET scan. With only a single patient study and only a broad comparison of SUV measurements, this work does not establish generalizability of clinical performance but primarily serves to demonstrate the feasibility of clinical imaging with the BPET–DBT scanner. While we did not observe noticeable randoms tail, suggesting minimal impact from random coincidences in this clinical study, the slightly higher BPET SUV compared to WB‐PET is likely not significant. The WB‐PET scan also highlights the challenging situation under which this scan was performed, as the lesion was located superiorly and posteriorly in the breast, in proximity to the patient's chest wall. Dedicated breast‐PET imaging has traditionally focused on only imaging the breast and has limited capability for imaging the axillary region of the breast. Addressing this limitation was not a primary objective for BPET–DBT, as we anticipate it will serve primarily in applications other than tumor detection and localized cancer staging.

The primary limitation of the scanner used in this study is its relatively small 5 cm axial FOV for BPET imaging, which arises from the use of a prototype coincidence logic board which supports communication with only 8 of the 16 detector modules, thereby limiting the electronically active axial FOV and also the overall scanner sensitivity. A recent MCU upgrade enables communication with all 16 detector modules and has expanded the axial FOV to 10 cm, permitting imaging of a larger portion of the breast with higher sensitivity. However, with this upgrade, the intrinsic detector design remains unchanged. Therefore, the performance characteristics reported in this study are expected to remain representative of the upgraded system, with improvements primarily reflecting the increased axial coverage and sensitivity. In addition, we have made progress toward implementing the gantry rotation mechanism needed to acquire multiple DBT views beyond the standard CC view. As we continue human imaging studies with the BPET–DBT scanner, this will support future imaging in the mediolateral‐oblique view, which is better suited for evaluating the upper‐outer quadrant of the breast.

## 5 CONCLUSION

In this work, we developed a dedicated breast PET scanner and integrated it with a digital breast tomosynthesis scanner. Besides providing anatomical images, the DBT scan also facilitates accurate PET attenuation correction. The BPET–DBT scanner can thus provide intrinsically co‐registered anatomical DBT images with high spatial resolution functional images from BPET. Detailed performance characterization of the BPET scanner, demonstrating its spatial resolution, imaging and quantification capabilities relevant to breast imaging, is presented along with the first human imaging study that demonstrate its readiness for clinical use. Pilot human imaging under the current protocol will continue in order to further investigate and refine the clinical utility of the scanner.

## CONFLICT OF INTEREST STATEMENT

Andrew D. A. Maidment is a shareholder and scientific board member of Real Time Tomography, LLC. No other potential conflict of interest relevant to this article is to be reported by the other authors.
